# Early Population Dynamics of “*Candidatus* Liberibacter asiaticus” in Susceptible and Resistant Genotypes After Inoculation With Infected *Diaphorina citri* Feeding on Young Shoots

**DOI:** 10.3389/fmicb.2021.683923

**Published:** 2021-06-09

**Authors:** Mônica Neli Alves, Juan Camilo Cifuentes-Arenas, Laudecir Lemos Raiol-Junior, Jesus Aparecido Ferro, Leandro Peña

**Affiliations:** ^1^Faculdade de Ciências Agrárias e Veterinárias (FCAV), Universidade Estadual Paulista (UNESP), Jaboticabal, Brazil; ^2^Fundo de Defesa da Citricultura (Fundecitrus), Araraquara, Brazil; ^3^Empresa Brasileira de Pesquisa Agropecuária (Embrapa), Cruz das Almas, Brazil; ^4^Instituto de Biología Molecular y Celular de Plantas (IBMCP), Consejo Superior de Investigaciones Científicas (CSIC), Universidad Politécnica de Valencia (UPV), Valencia, Spain

**Keywords:** HLB, Las multiplication, *Citrus* spp., *Murraya paniculata*, Bergera koenigii, bacterial growth, qPCR

## Abstract

Huanglongbing is a highly destructive citrus disease associated with “*Candidatus* Liberibacter asiaticus” (Las), a phloem−limited and non-culturable bacterium, naturally transmitted by the psyllid *Diaphorina citri.* Although diverse approaches have been used to understand the molecular mechanisms involved in the pathogen–host interaction, such approaches have focused on already infected and/or symptomatic plants, missing early events in the initial days post-inoculation. This study aimed to identify the time course of Las multiplication and whole-plant colonization immediately following inoculation by infected psyllids feeding for 2 days. Thus, the experimental approach was to track Las titers after psyllid inoculation in new shoots (NS) of *Citrus × sinensis* (susceptible), *Murraya paniculata* (partially resistant), and *Bergera koenigii* (fully resistant). Soon after psyllid removal, Las titers dropped until the 10–12th days in all three species. Following this, Las titers increased exponentially only in *C.* × *sinensis* and *M. paniculata*, indicating active bacterial multiplication. In *C. × sinensis*, Las reached a stationary phase at ∼5 log Las cells/g of tissue from the 40th day onward, while in *M. paniculata*, Las increased at a lower rate of up to ∼3 log Las cells/g of tissue between the 40th and 60th days, decreasing gradually thereafter and becoming undetectable from the 160th day onward. In *B. koenigii*, Las titers decreased from the start and remained undetectable. In *C. × sinensis*, an average of 2.6 log of Las cells/g of tissue was necessary for Las to move out of 50% of the NS in 23.6 days and to colonize the rest of the plant, causing a successful infection. Conversely, the probability of Las moving out of the NS remained below 50% in *M. paniculata* and zero in *B. koenigii*. To our knowledge, this is the first study on Las dynamics and whole-plant colonization during the earliest stages of infection. Identification of critical time-points for either successful multiplication or Las resistance may help to elucidate initial events of Las–host interactions that may be missed due to longer sampling intervals and at later stages of infection.

## Introduction

Huanglongbing (HLB) is the most severe citrus disease worldwide (Bové, 2006). In Asia and America, the unculturable α-proteobacteria, phloem-limited “*Candidatus* Liberibacter asiaticus” (Las) is associated with HLB (Gottwald, 2010). It is transmitted in the field by the Asian citrus psyllid *Diaphorina citri* (Kuwayama) (Sternorrhyncha: Liviidae) (Capoor et al., 1967). As there is no known cure for this disease, HLB control in Brazil is based on the use of healthy plants from certified nurseries, elimination of symptomatic plants in the groves, and inspection and rigorous control of the insect vector (Bassanezi et al., 2020). However, these strategies may be economically and environmentally unsustainable and inefficient for the management of HLB, whose incidence continues to increase, and it is currently estimated to be present in more than 20% of symptomatic trees in the citrus belt of São Paulo and Minas Gerais (Fundecitrus, 2020). In Florida, United States, the citrus industry relies on the production of more than 80% of the orchards that are Las-infected (Singerman and Useche, 2016). Leaves from affected trees show typical HLB symptoms such as asymmetrical chlorosis called blotchy mottle, enlarged veins, and intense canopy defoliation (Bové, 2006). The evolution of symptoms in Las-positive plants is rapid once they appear, and as the disease severity increases, the yield and fruit quality decrease (Gottwald, 2010). HLB-affected fruits are smaller, lighter, and highly acidic and have a reduced Brix ratio (Bassanezi et al., 2009).

*Diaphorina citri* acquires Las when they feed on the phloem sap from infected trees by inserting their stylets into the sieve elements. Las infects psyllids in a propagative and circulative manner (Inoue et al., 2009; Ammar et al., 2016; Canale et al., 2017). After a minimum latent period of 2–3 weeks, infected psyllids can transmit the bacterium to healthy plants by releasing it directly into the phloem when feeding (Canale et al., 2017). Electrical penetration graph (EPG) experiments have shown that 8 h is usually sufficient for the psyllid to come in contact with the phloem (Bonani et al., 2010; Wu et al., 2016; Carmo-Souza et al., 2020), but the efficiency of Las transmission is strongly dependent on the developmental stage of the plant tissue in which the insect settles as well as the environmental conditions (Raiol-Junior et al., 2017; Lopes and Cifuentes-Arenas, 2021; Pandey et al., 2021). The nature of insect saliva effectors capable of counteracting host defenses, similar to those shown for other phloem-feeding insects (Hogenhout and Bos, 2011), is largely unknown for *D. citri*. Since Las is released into the phloem, it is notable that symptoms appear in infected plants after the bacterium has already reached high titers, in a time period ranging from 4 to 8 months after inoculation under experimental conditions (Lopes et al., 2009) and to more than a year under field conditions (Gottwald, 2010). However, little is known about citrus host–Las interactions before the onset of symptoms, especially during the initial days and weeks after psyllid inoculation. Pandey and Wang (2019) detected Las as early as 2 days after *D. citri* fed on new shoots (NS), but the subsequent population growth was not monitored. It has been reported that Las moves through the phloem pores in an elongated form and adheres to the plasma membrane in sieve plates through unknown extracellular dark materials (Achor et al., 2020). Las preferentially invades new tissues during development (Raiol-Junior et al., 2021a) and irregularly colonizes different parts of the plant (Tatineni et al., 2008; Li et al., 2009), which makes the selection of the tissue used for study and/or diagnostics very important (Tatineni et al., 2008; Johnson et al., 2014; Raiol-Junior et al., 2021a). Moreover, losses in the root system caused by Las occur before the appearance of symptoms in the canopy (Johnson et al., 2014). Therefore, the first steps of the vector–plant–pathogen interaction are challenging to study. In addition, most previous studies were conducted using only susceptible *Citrus* species.

Although susceptibility to Las varies among *Citrus* species and varieties (Folimonova et al., 2009), full resistance has already been described in species that are sexually and/or graft-compatible with *Citrus* (Alves et al., 2021) as well as in species that are phylogenetically more distant within the family Rutaceae, subfamily Aurantioideae (Ramadugu et al., 2016; Beloti et al., 2018; Cifuentes-Arenas et al., 2019). *Murraya paniculata* (L.) Jack and *Bergera koenigii* L. plants, known as orange jasmine and curry leaf, respectively, are commonly used as ornamental plants in tropical and subtropical regions of the world (Swingle and Reece, 1967). Both these species are good hosts for *D. citri* (Damsteegt et al., 2010; Teck et al., 2011; Westbrook et al., 2011), being much more attractive to psyllid females as compared to sweet orange under laboratory and field conditions (Beloti et al., 2017; Tomaseto et al., 2019). However, low suitability and immunity to Las have been reported in *M. paniculata* and *B. koenigii*, respectively (Damsteegt et al., 2010; Beloti et al., 2018; Cifuentes-Arenas et al., 2019). The role of *D. citri* as a Las vector and the first stages of Las infection in the phloem of the two plant species remain unexplored.

We studied the time course of Las multiplication in the susceptible host *Citrus × sinensis* ‘Valencia’, in the transient host *M. paniculata*, and in the fully resistant *B. koenigii* to better understand the initial bacterial dynamics between the delivery of Las into the phloem by adults psyllids, bacterial multiplication at the first stages post-inoculation, and the time taken for Las to get out from the NS and spread through the rest of the plant. The use of a well-controlled challenge-inoculation system using infective psyllids allowed us to replicate experiments with consistent results, leading to the establishment of the earliest events of Las infection and whole-plant colonization. Understanding Las progression and defense responses triggered by resistant species may help to develop bacterial control strategies.

## Materials and Methods

### Plant Material and Growth Conditions

One-year-old plantlets of *Citrus* × *sinensis* (L.) Osbeck ‘Valencia’ sweet orange grafted on ‘Rangpur’ lime (*Citrus* × *limonia* Osbeck) rootstock and 1-year-old seedlings of *M. paniculata* (orange jasmine) and *B. koenigii* (curry leaf) were used. All plants were grown in 300-ml conical tubes (6.5 *×* 5.9 cm, upper *×* lower diameter; 16 cm, height), filled with coconut fiber, and irrigated twice a week. Prior to the experimental use, the *C.* × *sinensis* plantlets were 20 to 30 cm above the substrate level, and *M. paniculata* and *B. koenigii* were 20–40 cm above the substrate level. Rearing of Las-infected *Diaphorina citri* and studies on the time course of Las multiplication were carried out inside a controlled environmental room (CER) using shelves where the light source was 20 to 30 cm above the plants. The daily temperature varied from 24°C to 27°C, relative humidity ranged between 55% and 78%, and photoperiod was set to 12 h of light at 300 μmol m^–2^ s^–1^ of photosynthetically active radiation and 12 h of darkness. At the end of the longest sampling time (see below) for up to 12 months, plants were transferred from CER to a greenhouse in which the mean daily air temperature varied between 18.5°C and 34.4°C, and illumination was natural.

### Rearing of Las-Infective Psyllids and Inoculation Procedure

**FIGURE 1:**
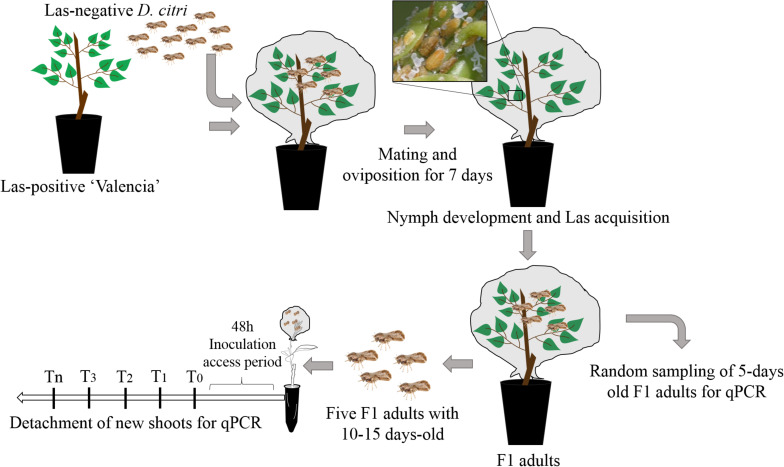
Experimental procedures for “*Candidatus* Liberibacter asiaticus” transmission by *Diaphorina citri.*

A methodology adapted from Lopes and Cifuentes-Arenas (2021) was used for insect rearing and inoculation purposes. Las-positive, fully symptomatic, and 2-year-old plants of *C. × sinensis* grafted on ‘Swingle’ citrumelo (*Citrus* × *paradisi* Macfad. × *Poncirus trifoliata* L. Raf.) growing in 4.7-l pots were used for rearing Las-infective *D. citri* insects. The Ct value of Las in these source plants was 20.7 ± 0.33 (equivalent to ca. 6.2 ± 0.1 log of Las cells/g of tissue). Las infection in *C. × sinensis* plants was confirmed by qPCR (Li et al., 2006). Plants were lightly pruned to stimulate the growth of NS, and when they reached the V2 stage of development (Cifuentes-Arenas et al., 2018), four Las-negative 10- to 20-day-old psyllids were confined per shoot for oviposition for 7 days. The Las-negative mixed sex psyllids were obtained from a colony kept at Fundecitrus, reared on healthy *M. paniculata*, adapting the methodology proposed by Skelley and Hoy (2004). The adult psyllids were then removed from the plants, and the plants remained inside the CER to allow for the eggs to hatch and nymphs to develop into first-generation adults (F1). Once F1 adults began to emerge, a random sample of three insects per source plant was taken at 5 days postemergence to confirm Las presence by qPCR, and the rest were maintained in the same plants up to 10–15 days before being used (Figure 1).

**FIGURE 2:**
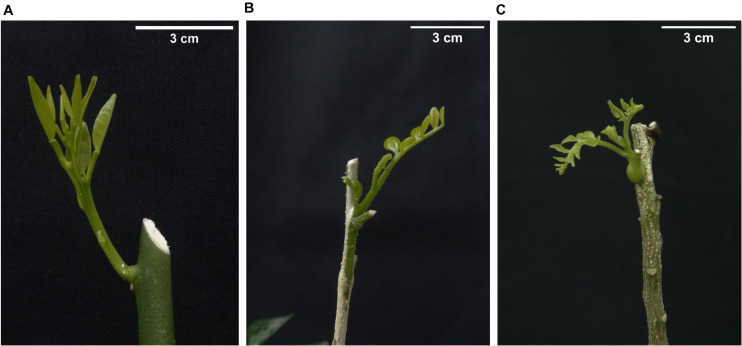
Aspect of new shoots in **(A)** *Citrus × sinensis*, **(B)** *Murraya paniculata*, and **(C)** *Bergera koenigii.* Such shoot stages were used for “*Candidatus* Liberibacter asiaticus” challenge *Diaphorina citri*-mediated inoculation experiments.

The *C.* × *sinensis* plantlets and the *M. paniculata* and *B. koenigii* seedlings were pruned at ∼15 cm above the substrate level to force NS growth until they reached stage V3 of development 18 to 23 days post-pruning (Cifuentes-Arenas et al., 2018). In *M. paniculata* and *B. koenigii*, V3 shoots were characterized by the presence of one partially unfurled and one or more tender expanding leaflets (Figure 2).

Five 10–15-day-old Las-exposed adults were confined to a single V3 NS per plant for 48 h of inoculation access period (IAP). The adults were then removed and stored at −20°C. Subsequently, random-stratified samplings were performed, where one of the five adult insects from each of three randomly selected plants per NS detaching time (see below) for qPCR analysis was selected. The plants were then sprayed with insecticide (Abamectin EC 7.2 g of active ingredient per 1 l of water) to eliminate any psyllid eggs or nymphs present. This experiment was repeated twice with 70, 100, and 41 individuals of *C. × sinensis*, *M. paniculata*, and *B. koenigii*, respectively, in the first replication, and 95, 106, and 70 individuals in the second replication. As a control group, 8–10 plants of each species in both experimental replicates were exposed to Las-negative adults of similar age and conditions as described above.

### Time Course of Las Multiplication and Whole Plant Colonization

To determine the population dynamics of Las multiplication after psyllid feeding, 5–10 plants per genotype were randomly selected at each time point, and the NS on which the Las-positive adults fed was detached from time 0 (day of psyllid removal) and at variable times post-IAP. Each plant was used at a single time point. For *C. × sinensis*, sampling was performed at 0, 5, 10, 15, 25, 40, 55, and 70 days and 0, 5, 10, 15, 18, 20, 30, 40, 65, and 95 days post-IAP in the first and second experiment replicates, respectively. For *M. paniculata*, sampling was performed at 0, 5, 10, 15, 25, 40, 55, 70, 95, 110, and 180 days and 0, 5, 10, 15, 20, 25, 30, 40, 65, 95, and 170 days post-IAP in the first and second experiment replicates, respectively. For *B. koenigii*, sampling was performed at 0, 5, 10, 15, 30, 60, and 95 days and 0, 5, 10, 15, 20, 34, and 55 days post-IAP in the first and second experiment replicates, respectively. NS was analyzed to verify the presence of Las by qPCR. In the first sampling intervals, the whole NS on which the Las-positive adults fed was used for processing, but as they grew, only a proportion of the tissue was necessary; it was ensured that tissue pieces were randomly sampled from all parts (tip to base) of the NS on which the Las-positive adults fed. At the end of the longest sampling time (180 days), the plants were transferred from the CER to a greenhouse to complete up to 12 months post-IAP. Then, these plants were sampled again, selecting four to five leaves randomly distributed in the whole canopy. The entire leaves were processed to detect Las presence using qPCR; thus, the successful establishment of the infection was verified.

### DNA Extraction and qPCR

Tissue samples (0.3 g) were processed as described by Teixeira et al. (2005) using a TissueLyser II system (Qiagen, Valencia, CA, United States) at 45 Hz for 30 s in a 2.0-ml microtube containing 5-mm steel beads. For psyllid DNA extraction, one insect and a 3-mm steel bead were used in a 1.5-ml microtube. Total DNA was extracted using the cetyltrimethylammonium bromide (CTAB) extraction buffer as mentioned in Murray and Thompson (1980), with adaptations. Briefly, for *C. × sinensis*, *M. paniculata*, and *D. citri* DNA extraction, 1.5 ml of CTAB buffer was used (2% CTAB; 1.4 M NaCl; 2% PVP 10000; 0.5 M EDTA pH 8; 1 M Tris–HCl pH 8; 0.2% β-mercaptoethanol), incubated at 65°C for 30 min, and then centrifuged for 10 min at 12000 *× g*. The supernatant was transferred to a new 1.5-ml microtube, followed by extraction with chloroform/isoamyl alcohol (24:1). For *B. koenigii*, to avoid oxidation of phenolic compounds after sampling, 0.3 g of the tissue was immediately cooled with liquid nitrogen and stored at −80°C until DNA extraction. The tissue trituration was done with 350 μl of a modified CTAB buffer (2% CTAB; 1.4 M NaCl; 2% PVP 10000; 0.5 M EDTA pH 8; 1 M Tris–HCl pH 8; 5% β-mercaptoethanol) and then 1.15 ml of this buffer was added, incubated at 60°C for 20 min, and centrifuged for 10 min at 12000 *× g*. The supernatant was transferred to a new 1.5-ml microtube, followed by two extractions with phenol/chloroform/isoamyl alcohol (25:24:1) and one with chloroform/isoamyl alcohol (24:1). In both extractions, DNA precipitation was performed with 0.6 V isopropanol, washed twice with 70% ethanol, and resuspended in 50 μl of Milli-Q^®^ water. DNA quality was checked using a NanoDrop (Thermo Fisher Scientific, Massachusetts, EUA) (Desjardins and Conklin, 2010).

For real-time polymerase chain reactions (qPCR) and the identification of the 16S rRNA of Las, 1.0 μl of total DNA (100 ng/μl), TaqMan^®^ PCR Master Mix (1×) (Invitrogen, Carlsbad, CA, United States), and HLBas primer/probe (0.5 μM/0.2 μM) were used in a StepOnePlus thermocycler (Applied Biosystems, California, USA) as described by Li et al. (2006). To estimate Las multiplication in time-course experiments, qPCR analysis was performed in triplicate, and for Las colonization and Las detection in the whole plant, qPCR analyses were performed only once. The mitochondrial gene *cytochrome oxidase* (*COX*) and an insect *wingless* (*Wg*) gene region were used in all samples as internal controls for the quality of plants and psyllid DNA, respectively (Li et al., 2006; Manjunath et al., 2008). In addition, negative and positive citrus leaf and psyllid samples were included during DNA extraction and qPCR assays. Las was quantified based on the linear relationship between Ct and the 16S rRNA log, according to Lopes et al. (2013). As Ct values higher than 34.0 (below 0.9 log Las cells/g of tissue) produce variable results (Lopes et al., 2013), samples were considered Las-positive when the qPCR cycle threshold (Ct) was lower than 34.0, suspected to be positive when Ct values were between 34.0 and 36.0, and negative when Ct > 36 and/or when undetermined.

### Data Analysis

Data on adult survival at the time of psyllid removal were analyzed using a chi-square test by adjusting a binomial generalized linear model (GLM). To determine if there were significant differences in Las titers between the groups of psyllids used to inoculate the three plant species (in each repetition of the experiment and between time points within each species), a two-way ANOVA with interactions was first performed considering the species and the repetition of the experiment as a factor with three and two levels, respectively. Subsequently, the possible differences between the time points within each species were analyzed by performing a one-way ANOVA, considering each shoot detachment time point as a treatment. These analyses were performed by adjusting a Gaussian GLM. Similarly, to compare the initial bacterial load in NS between species at the time of psyllid removal, a two-way ANOVA (plant species × experiment repetition) was carried out. When significant differences were found, the means were separated using Tukey’s post hoc test.

To analyze the time course of Las population multiplication in NS after psyllid removal, data on Las titer were submitted to nonlinear regressions to compare several growth models. The best model was selected based on the overall model significance and *R*^2^. For this, data consisted of log values estimated over the Ct values (including those above 34) according to Lopes et al. (2013). An arbitrary value of 39.9999 was assigned to the qPCR results that gave “undetermined” outputs.

Data on new shoot growth were subjected to nonlinear regression to adjust the Gompertz growth model (Tjørve and Tjørve, 2017). To analyze the probability of successful infection after 48 h of psyllid IAP and subsequent detachment of NS at different time intervals, data on Las-positive leaf samples (Ct < 34.0) taken 1 year after the start of the experiments were subjected to a logistic regression by adjusting a binomial GLM (Peng et al., 2002).

## Results

**FIGURE 3:**
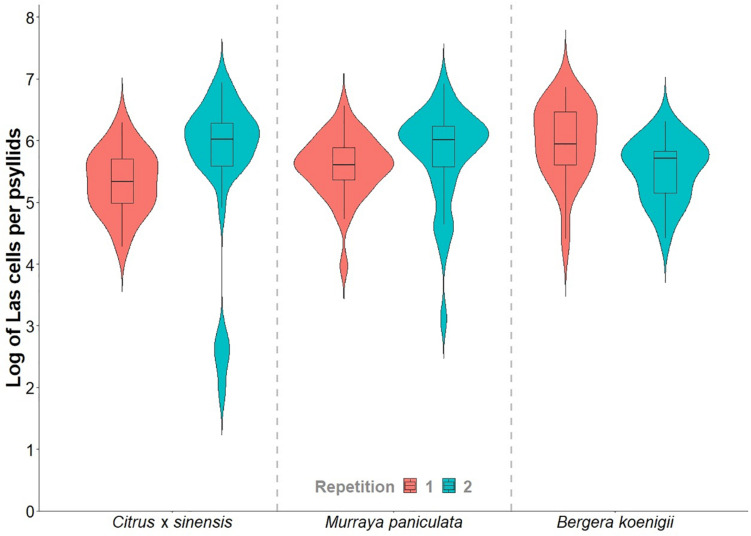
Violin plots of the “*Candidatus* Liberibacter asiaticus” titer in a subsample of 15- to 20-day-old *Diaphorina citri* adults reared on Las-positive *Citrus × sinensis* plants and confined during 48 h of inoculation access period (IAP) in new shoots of healthy *Citrus × sinensis*, *M. paniculata*, and *B. koenigii*.

Adult survival after 48 h of IAP was not significantly different between plant species [*χ^2^_(2, 2403)_* = 466.81; p > 0.05]. On an average, psyllid survival after the confinement period was 99.1%, 99.6%, and 96.6% in the first experiment and 95.8, 97.4, and 99.4% in the second experiment replicates on *C. × sinensis* ‘Valencia’, *M. paniculata*, and *B. koenigii*, respectively (Supplementary Table 1). Las titers in psyllids did not differ between plant species on which they were confined [*F*_(2,158)_ = 1.4863; *p* > 0.05] or between replicates [*F*_(1,158)_ = 0.1535; *p* > 0.05], and there was no significant interaction [*F*_(2,158)_ = 2.6445; *p* > 0.05] between these two factors (Supplementary Table 2). At different time points after NS detachment, there was no difference in Las titers in psyllids used in *C. × sinensis* [*F*_(12,53)_ = 0.9967; *p* > 0.05], *M. paniculata* [*F*_(14,64)_ = 1.7563; *p* > 0.05], and *B. koenigii* [*F*_(10,38)_ = 1.7061; *p* > 0.05]. The averages of Las titers varied from 5.33 to 5.98 log (min. = 1.94; max. = 6.94; Figure 3). The amount of initial bacterial load injected by the adult psyllids after 48 h of IAP was not influenced by the plant species [*F*_(2,50)_ = 0.003; *p* > 0.05] or by the repetition of the experiment [*F*_(1,50)_ = 1.542; *p* > 0.05]. The interaction between the two factors was not significant [*F*_(2,50)_ = 3.220; *p* = 0.05]. Details on Ct and log values of each plant and time of evaluation are shown in Supplementary Tables 3, 4.

**FIGURE 4:**
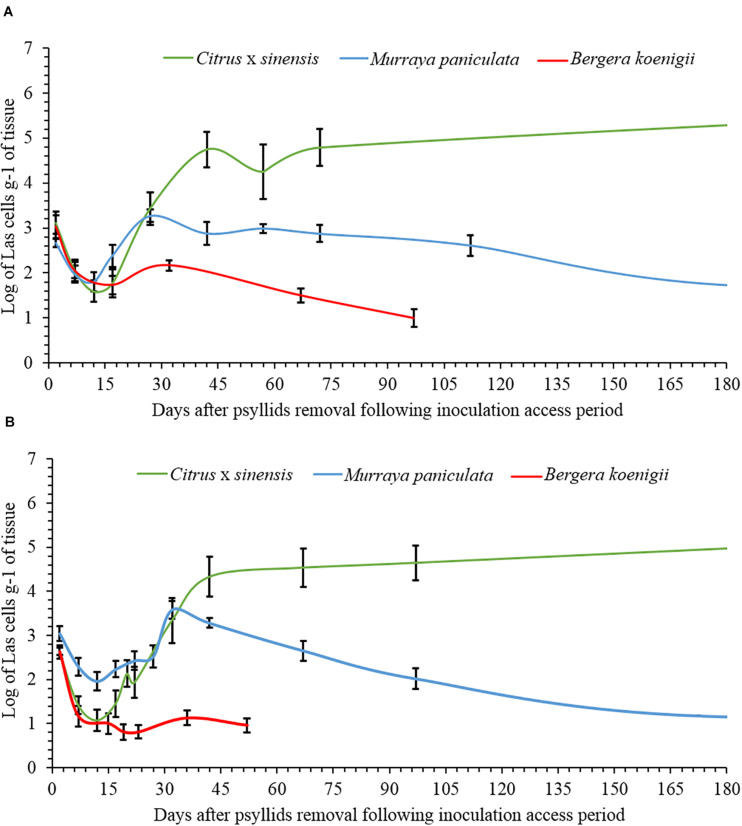
Evolution of the population dynamics of “*Candidatus* Liberibacter asiaticus” over time in new shoots of *Citrus × sinensis*, *Murraya paniculata*, and *Bergera koenigii*, following a 48-h period of Las-exposed *Diaphorina citri* confinement in experiment replicates 1 **(A)** and 2 **(B)**.

The data on Las titers in *C. × sinensis* and *M. paniculata* adjusted well to the Lorentzian growth model (Table 1 and Supplementary Figure 1). This model describes two contrasting situations for both species in both replicates (Figure 4). At time 0 (the day of psyllid removal after 48 h of IAP), 10/10 and 7/10 *C. × sinensis* plants had Ct values lower than 34.0, in the first and second replicates, respectively. The average Las titer in NS at time 0 was 2.91 ± 0.16 log cell/g of tissue. After this, there was an initial decrease in Las titers soon after psyllid removal until the 10–12th days, up to ∼2.0 log cell/g of tissue. Subsequently, Las multiplied exponentially, reaching ∼90% of its final population after ∼40 days (Figure 4). A stationary phase was then observed, and the bacterial titer remained stable (∼4.5–5 log cell/g of tissue) until the last evaluation (70 and 95 days post-IAP in experiment replicates 1 and 2, respectively) with 80%–100% Las-positive plants.

**TABLE 1:** Nonlinear regression models describing the time course of “*Candidatus* Liberibacter asiaticus” population dynamics in new shoots after 48 h of Las-exposed *Diaphorina citri* confinement at 25°C ± 2°C.

	Model parameters				Model statistics		
	β_0_ ± SE	β_1_ ± SE	β_2_ ± SE	β_3_ ± SE	*F*_(df)_	*P(> F)*	*R*^2^
Replicate 1							
*C.* × *sinensis*^*a*^	5.356 ± 0.333	13.343 ± 1.730	29.682 ± 7.507	−176.41 ± 47.940	24.85_(3, 9)_	0.002	0.8994
*M. paniculata*^*a*^	1.389 ± 0.418	64.819 ± 12.341	130.6 ± 57.449	339.349 ± 201.587	4.92_(3, 11)_	0.0381	0.5403
*B. koenigii*^*b*^	3.424 ± 0.445	−0.211 ± 0.051	–	–	105.73_(2, 6)_	< 0.001	0.7627
Replicate 2							
*C.* × *sinensis*^*a*^	5.172 ± 0.220	12.798 ± 1.044	32.917 ± 5.326	−206.907 ± 34.916	69.42_(3, 11)_	< 0.001	0.9535
*M. paniculata*^*a*^	1.118 ± 0.388	45.641 ± 6.797	84.272 ± 32.31	277.056 ± 125.897	6.66_(3, 12)_	0.0126	0.6207
*B. koenigii*^*b*^	3.285 ± 0.488	−0.405 ± 0.073	–	–	76.80_(2, 7)_	< 0.001	0.8018

*^*a*^The final model selected was $Las=\beta_{0}+\frac{2\beta_{3}}{\pi }\frac{\beta_{2}}{4{\left(x-\beta_{1}\right)}^{2}{\left(\beta_{2}\right)}^{2}}$, were β_0_ is the baseline offset, β_1_ is the center of the peak, β_2_ is the full width of the peak at half height, and β_3_ is the total area of the curve from the baseline. ^*b*^The final model selected was Las β_0_x^β_1_^, were β_0_ is the coefficient and β_1_ is power.*

Moreover, 9/10 and 10/10 *M. paniculata* plants were Las-positive at time 0 in the first and second experiment replicates, with an average titer of 2.90 ± 0.10 log cell/g of tissue. The initial decrease in Las titer up to the 10–12th days was less severe than that in *C. × sinensis*, with an average titer of ∼2.5 log cell/g of tissue. The subsequent exponential increase was also less pronounced, and Las reached a maximum titer between the 40th and 60th days, with a population similar to that of the day of psyllid removal (∼3 log cell/g of tissue). After reaching this maximum, Las titer decreased slowly until it reached undetectable levels from the ∼180th and 170th days onward in the first and second experiment replicates, respectively (Figure 4).

In *B. koenigii*, the best model describing Las population progress was the negative exponential model (Table 1 and Supplementary Figure 1). At time 0, 6/8 and 6/10 *B. koenigii* plants showed Ct values lower than 34.0, in the first and second replicates, respectively. The average of the Las titer in NS at time 0 was 2.93 ± 0.15 log cells/g of tissue, but after this, a fast reduction in Las titer following psyllid removal was observed, reaching undetectable levels from the 30th and 10th days onward in the first and second replicates, respectively (Figure 4).

**FIGURE 5:**
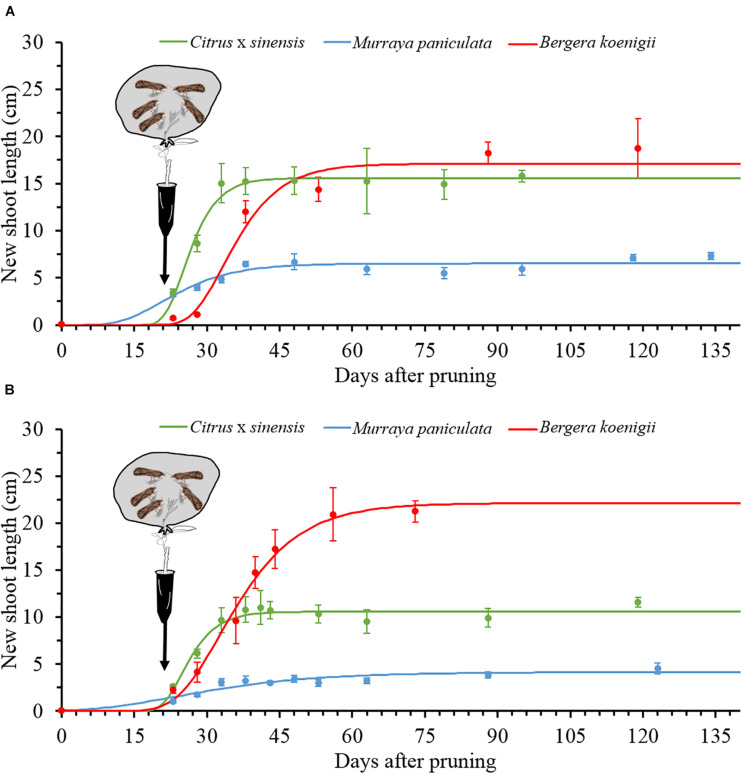
The Gompertz models for new shoot growth of *Citrus × sinensis*, *M. paniculata*, and *B. koenigii* in an acclimatized room at 25°C (black arrows indicate the day of psyllid confinement for a 48-h inoculation access period (IAP), (see section “Materials and Methods” for details), in experiment replicates 1 **(A)** and 2 **(B)**.

For the three species, the Gompertz model described sufficiently well the growth of NS (Table 2), with very active growth from 15 to approximately 45 days after pruning. The maximum rate of NS growth was found in *C. × sinensis* at days 25–30 in both experimental replicates, followed by *B. koenigii* at days 33–35 also in both experimental replicates. *M. paniculata* presented the lowest growth rate, reaching the maximum rate at days 20 and 23 in replicates 1 and 2, respectively (Figure 5 and Supplementary Table 5).

**TABLE 2:** Parameters of the Gompertz growth model of new shoots of *Citrus × sinensis*, *Murraya paniculata*, and *Bergera koenigii*.

	Model parameters			Model statistics		
	β_0_ ± SE	β_1_ ± SE	β_2_ ± SE	*F*_(df)_	*P(> F)*	*R*^2^
Replicate 1						
*C.* × *sinensis*^*a*^	15,586 ± 0,872	25,027 ± 0,99	0,255 ± 0,082	157.18_(3, 77)_	< 0.001	0.5711
*M. paniculata*^*a*^	6,52 ± 0,214	20,53 ± 2,068	0,123 ± 0,037	483.95_(3, 107)_	< 0.001	0.6226
*B. koenigii*^*a*^	17,103 ± 0,814	33,345 ± 1,268	0,153 ± 0,034	221.61_(3, 41)_	< 0.001	0.8922
Replicate 2						
*C.* × *sinensis* ^*a*^	10,579 ± 0,447	24,697 ± 0,922	0,249 ± 0,074	271.25_(3, 102)_	< 0.001	0.5606
*M. paniculata*^*a*^	4,139 ± 0,221	23,431 ± 2,57	0,062 ± 0,015	316.01_(3, 113)_	< 0.001	0.5615
*B. koenigii*^*a*^	22,132 ± 1,599	32,672 ± 1,434	0,11 ± 0,024	179.62_(2, 77)_	< 0.001	0.6964

^a^The final model selected was Shootsize = β_0_*e^(−e^(−β_2_*(time−β_1_))^)^, where β_0_ is maximum shoot size (cm), β_1_ is the center of the curve (days), and β_2_ is the growth rate (cm day^−1^).

**FIGURE 6:**
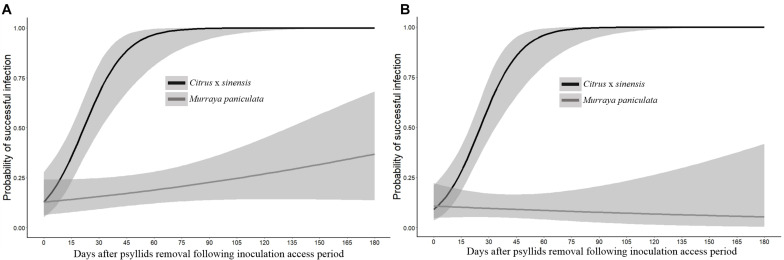
Probability of occurrence of a successful infection of the whole plant of *Citrus × sinensis* and *M. paniculata* after an initial 48 h of Las-exposed psyllid confinement and subsequent removal of new shoots at different time intervals in experiment replicates 1 **(A)** and 2 **(B)**.

In *C. × sinensis*, the time after 48 h of IAP in which NS was detached significantly affected the probability of a successful infection in the whole plant, evaluated by qPCR 1 year after the initial insect confinement (Table 3), indicating that Las was restricted to the NS during the first weeks post-inoculation. In *C. × sinensis*, the mean time for the NS to begin to serve as source of Las for the rest of the plant and thus to establish a successful infection was 21.8 days (∼15 to 30 days) in the first experiment replicate and 25.3 (∼18 to 33 days) in the second replicate. For *M. paniculata*, the results indicated that Las was almost unable to achieve whole-plant stable infections in the host (Figure 6), as only 18 out of 100 and 10 out of 106 seedlings in the first and second experiment replicates, respectively, were infected out of the NS at 12 months after psyllid inoculation, and such Las-positive plants were distributed randomly along the time points evaluated in both replicates (Supplementary Table 4). In *B. koenigii*, Las could not establish successful infections regardless of the NS detachment time.

**TABLE 3:** Logistic models for the probability of a successful infection after 48 h of Las-positive inoculation by *Diaphorina citri* adults onto new shoots.

	Model parameters^*a*^		Model statistics		
Species	*β_0_* ± SE	*β1* ± SE	*X*^2^_(df)_	*P(> X^2^)*	pseudo-*R*^2^
Replicate 1					
*C.* × *sinensis*	−**1.917 ± 0.494**	**0.088 ± 0.022**	67.25_(1, 68)_	5.40 × 10^–8^	0.3054
*M. paniculata*	−**1.920 ± 0.391**	0.008 ± 0.005	92.01_(1, 98)_	0.1322	0.0240
Replicate 2					
*C.* × *sinensis*	−**2.305 ± 0.503**	**0.091 ± 0.021**	88.06_1, 88)_	4.21 × 10^–9^	0.2816
*M. paniculata*	−**2.110 ± 0.438**	0.004 ± 0.008	65.97_(1, 104)_	0.6058	0.0040

*^*a*^SE, standard error of the parameter; bold values are significant (*p* < 0.05).*

## Discussion

In this study, we investigated the earliest steps of *D. citri*–Las–host interactions, which resulted in the establishment of either bacterial infection in the susceptible *C.* × *sinensis* ‘Valencia’ host or in resistance to Las in *M. paniculata* and *B. koenigii*. These two latter species were considered among the most suitable *D. citri* hosts among the Rutaceae, because volatile and visual cues made them more attractive than *Citrus* (Beloti et al., 2017; Tomaseto et al., 2019). The number of adult psyllids settled on the seedlings, number of eggs laid, and nymphs developed were also higher in *M. paniculata* and *B. koenigii* than in *Citrus* in host choice assays (Teck et al., 2011). However, we did not find differences among these three host plants in the ability of infected psyllids to inoculate Las into them, because Las showed similar titers at time 0 in NS of the three plant species, after the infected insects fed on them for 48 h. Although it has been reported that Las infection negatively affects *D. citri* performance (Ghanim et al., 2016) and induces important transcriptome reprogramming in the insect midgut (Yu et al., 2020), most infected *D. citri* individuals used in our experiments remained alive after 48 h of IAP and were efficient in transmitting Las to the three hosts. Morphological, developmental, and chemical differences among the three Rutaceae species (Beloti et al., 2018; Cifuentes-Arenas et al., 2019; Tomaseto et al., 2019) did not influence the ability of psyllids to overcome physical barriers and access sieve elements through their stylets to release the bacterium at relatively high titers. However, it is possible that Las acquisition by the psyllids may differ between these three host plants, whether due to the above differences or for other reasons including different Las titers in these hosts.

Once released into the phloem, bacterial titers decreased during the first 10–12 days after inoculation, reaching very low concentrations in the three species investigated. Such bacterial titer decline may be attributed, at least in part, to a dilution effect due to active NS growth a few days after pruning at the V2–V3 developmental stages in *C.* × *sinensis*, according to Cifuentes-Arenas et al. (2018). However, there was a clear lag between Las titer fall and NS enlargement, which occurred from 15 to 45 days after pruning. Moreover, Las decline was similar in the three species and quite comparable in the two replicates, while NS development followed slightly differing patterns in the two replicates, especially for *C. × sinensis* and *B. koenigii* (Figures 4, 5). Las has a reduced genome size and lacks genes involved in type 3 and most part of type 2 and 4 secretion systems, as well as those required for life *in vitro* (Duan et al., 2009). Indeed, it is only able to metabolize some sugars, which makes it dependent on phloem sap as a carbon source (Fagen et al., 2014), as it occurs in many other phloem-restricted bacteria (Huang et al., 2020). It is possible that Las needs some time to adapt to the new phloem environment to sequester metabolites from the host required to live and multiply. On the other hand, such initial bacterial titer decline does not occur when *Liberibacter crescens* strain BT-1, the only cultured wild-type strain of the *Liberibacter* genus, is grown *in vitro* under optimal nutritional and environmental conditions (Sena-Vélez et al., 2019). This suggests that the phloem does not provide the necessary metabolites to Las so fast and/or they need to be transformed for use by bacterial cells, as it occurs with sugars for spiroplasmas (André et al., 2005). Killiny (2016) proposed that the *M. paniculata* and *B. koenigii* phloem sap has poor nutrient qualities, which may be associated with the limited or lack of Las multiplication in these two species observed in later time-course phases, but not during the first 12 days after inoculation, because the bacterial titer decrease was similar in *C. × sinensis*, an excellent Las host.

Alternatively, bacterial titer decrease may be caused as a consequence of defense responses induced by plant cells to either psyllid damage through herbivore-associated molecular patterns or to Las through microbe-associated molecular patterns (Huang et al., 2020). Although it is unclear whether the latter occurs in phloem sieve elements (Jiang et al., 2019), it has been recently found that Las can attach and move into nucleated cells associated with the phloem, at least in seed coat phloem tissue (Achor et al., 2020), which would likely activate immune cell responses. Moreover, such a drastic and consistent decline in bacterial titers in different plant species at a few days post-inoculation may explain, at least in part, the relatively low efficiency of Las inoculation and the frequent failures in attempts to inoculate Las into citrus plants through psyllids under controlled conditions (Pelz-Selinski et al., 2010; Ammar et al., 2016; Canale et al., 2017). Therefore, Las should be able to overcome such a lag, probably caused by the presence of physical and chemical obstacles, to remain alive and proceed with the infection process. It is known that the Las genome encodes a functional salicylic acid hydroxylase capable of degrading salicylic acid to neutralize plant defenses (Li et al., 2017). Moreover, the Las prophage gene SC2-gp095 encodes a ROS-scavenging peroxidase, which may assist Las in evading plant defense responses (Jain et al., 2015).

After the decrease phase, the Las concentration increased exponentially until it reached a titer of approximately 5 log Las cells/g of NS tissue at 45 days, which was maintained stationary in the susceptible *C. × sinensis* host. Other studies conducted with already Las-infected plants, inoculated by grafting, obtained similar results in terms of stationary phase titers in susceptible citrus genotypes (Hilf and Luo, 2018; Raiol-Junior et al., 2021a). However, in our study, there was a lag of approximately 15–30 days for Las to reach a plateau of 5 log Las cells/g of tissue compared to the previous studies, which may be due to differences in the initial inoculum titers (budwoods vs. psyllids), a dose–effect already reported (Raiol-Junior et al., 2017), which seems to lose relevance once Las is established (Cifuentes-Arenas et al., 2019). An average of 23.6 (16.5–31.5) days of post-IAP was required for Las to move out of 50% of the NS and colonize the rest of the plant, causing a successful infection, that is, for the initially infected shoot to serve as a source of Las for the rest of the *C.* × *sinensis* plant. This occurred several months before Las-secreted proteins may be suitable for interaction with host cell enzymes to induce disease symptoms (Clark et al., 2018; Pang et al., 2020), including conspicuous callose deposition on sieve plates and consequent leaf mottling in infected *C.* × *sinensis* plants (Kim et al., 2009; Achor et al., 2010). Other phloem responses arising just after Las phloem translocation from infected tissues, but much earlier than the expression of symptoms, remain poorly understood.

In *M. paniculata*, Las population dynamics were similar to those of *C. × sinensis* up to the 30th day. Las reached a semi-stationary phase for about 15–30 days, and then bacterial titers declined progressively to become undetectable at 180 days onward. Moreover, Las translocation from the NS to the rest of the plant was rare and uneven. In previous controlled experiments with *M. paniculata*, the earliest samplings were taken from the second to fourth months post-IAP (Damsteegt et al., 2010; Cifuentes-Arenas et al., 2019). However, the continuous decrease in Las titers to undetectable levels confirms previous consideration of *M. paniculata* as a transient host for Las (Damsteegt et al., 2010; Cifuentes-Arenas et al., 2019). Even at low titers, the transitory presence of Las in *M. paniculata* (Damsteegt et al., 2010; Walter et al., 2012; Cifuentes-Arenas et al., 2019) suggests that this species may be a suitable host for Las spread. However, Las transmission from *M. paniculata* to *C. × sinensis* by *D. citri* was observed at very low frequencies (less than 1%) in controlled experiments and only when environmental and plant developmental conditions were highly favorable for Las infection (Cifuentes-Arenas et al., 2019). Indeed, both the transient nature of Las infection and its low titers when the bacterium remains detectable in this host explain the 43% reduction in HLB incidence in a sweet orange orchard when *M. paniculata* was used as a trap crop fence to efficiently attract and control the *D. citri* population and its spread (Tomaseto et al., 2019).

In contrast, Las could not be established in NS of *B. koenigii*, as shown previously by Beloti et al. (2018). However, we were able to detect Las multiplication in *B. koenigii* NS in one of the experiment replicates, indicating that under specific, highly suitable conditions, the bacterium could attain low but clearly detectable concentrations in the inoculated shoots, although only occasionally. Moreover, the probability of Las moving out of the NS was null in the *B. koenigii*. Whether *M. paniculata* and *B. koenigii* phloem sap are unsuitable for Las survival (Killiny, 2016) and/or their cells activate efficient defense responses to hold bacterial infections remains to be further investigated. Nevertheless, *B. koenigii* is more efficient than *M. paniculata* in limiting Las. Considering the different responses of Las infection between *Murraya paniculata* and *Bergera koenigii*, their close phylogenetic relationships, and the wide diversity of *Murraya* and *Bergera* species found in Asia (Nguyen et al., 2019), the interactions of the two genera with Las is worth investigating, as they seem to represent hosts at the frontier between resistance and susceptibility to the bacterium.

This is the first study to investigate the early population dynamics of Las in three species with contrasting susceptibility/resistance responses to this bacterium, by simulating the natural Las inoculation system using infective *D. citri* and evaluating the presence of Las in NS by qPCR at different short time intervals post-IAP. Using this controlled and reproducible system, we were able to expose all the plants to similar initial inoculum pressure, as they received the same initial bacterial load injected into the phloem by psyllids and the environmental conditions were adequate to maximize the chances of successful infection occurrence (Raiol-Junior et al., 2021b). Our results allowed us to identify the key time points of Las growth in plant genotypes with different responses to Las infection. This information could be essential to investigating whether or when plant cell defense responses are activated after wounding and bacterial release into the phloem by *D. citri*, as well as how Las is able to evade such responses in *C.* × *sinensis* but not in *B. koenigii* and only partially in *M. paniculata*. It may also help to identify suitable targets to be knocked out or overexpressed biotechnologically with the aim of creating HLB-resistant varieties.

## Data Availability Statement

The original contributions presented in the study are included in the article/Supplementary Material, further inquiries can be directed to the corresponding author.

## Author Contributions

MA, LP, and JF conceptualized the work. MA, LP, JF, and J-CA designed the work. MA, J-CA, and LR-J collected the data. J-CA performed the statistical analysis. MA, J-CA, LR-J, JF, and LP contributed to interpretation, drafting the article, and critical revision of the manuscript. All authors contributed to the article and approved the submitted version.

## Conflict of Interest

The authors declare that the research was conducted in the absence of any commercial or financial relationships that could be construed as a potential conflict of interest.
