# Supplementary material for: Early Population Dynamics of “Candidatus Liberibacter asiaticus” in Susceptible and Resistant Genotypes After Inoculation With Infected Diaphorina citri Feeding on Young Shoots
Source: Front Microbiol. 2021 Jun 9;12:683923. doi: 10.3389/fmicb.2021.683923 (PMC8219961; doi:10.3389/fmicb.2021.683923)
Supplement: Supplementary file 1 [file Image_1.pdf]

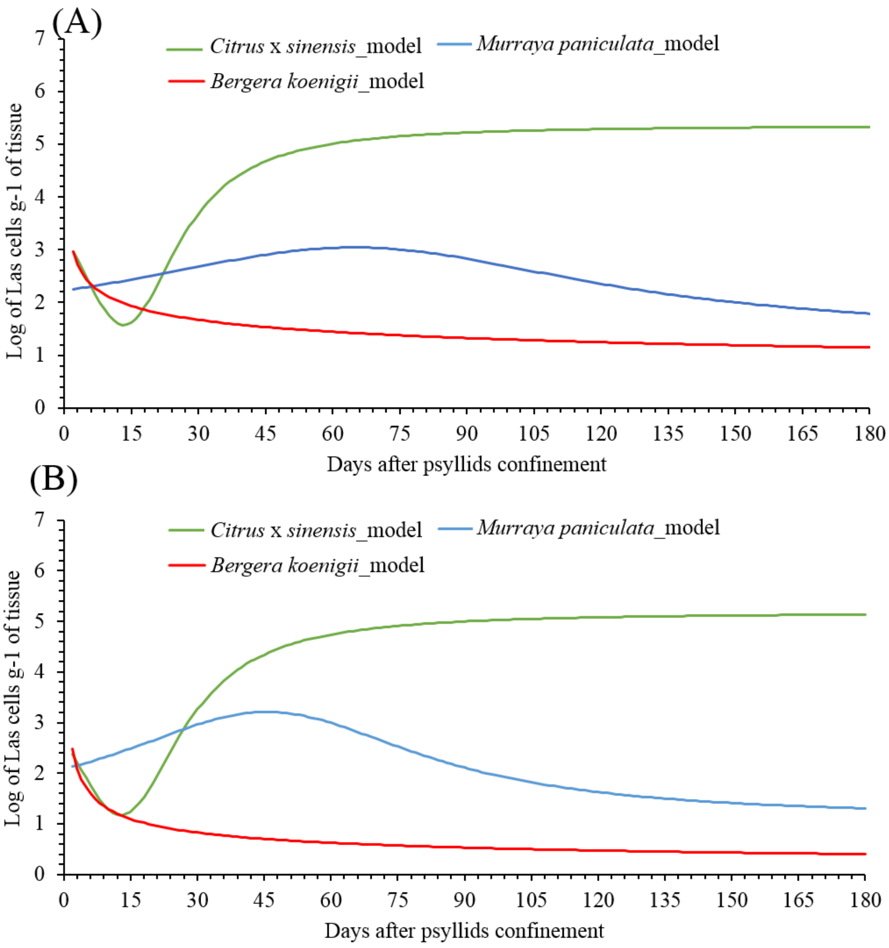

Supplementary Figure 1. Lorentzian growth model for the population dynamics of '*Candidatus* Liberibacter asiaticus' over time in new shoots of *Citrus* × *sinensis* and *Murraya paniculata* and ~~the~~ negative exponential model for the population dynamics of '*Candidatus* Liberibacter asiaticus' over time in new shoots of *Bergera koenigii*, following a 48 h period of *Diaphorina citri* confinement in experiment replicates 1 (**A**) and 2 (**B**).
